# Gap Junction Protein Connexin43 Exacerbates Lung Vascular Permeability

**DOI:** 10.1371/journal.pone.0100931

**Published:** 2014-06-26

**Authors:** James J. O’Donnell, Anna A. Birukova, Eric C. Beyer, Konstantin G. Birukov

**Affiliations:** 1 Section of Pulmonary and Critical Care, Department of Medicine, University of Chicago, Chicago, Illinois, United States of America; 2 Lung Injury Center, Department of Medicine, University of Chicago, Chicago, Illinois, United States of America; 3 Department of Pediatrics, University of Chicago, Chicago, Illinois, United States of America; Northwestern University Feinberg School of Medicine, United States of America

## Abstract

Increased vascular permeability causes pulmonary edema that impairs arterial oxygenation and thus contributes to morbidity and mortality associated with Acute Respiratory Distress Syndrome and sepsis. Although components of intercellular adhesive and tight junctions are critical for maintaining the endothelial barrier, there has been limited study of the roles of gap junctions and their component proteins (connexins). Since connexins can modulate inflammatory signaling in other systems, we hypothesized that connexins may also regulate pulmonary endothelial permeability. The relationships between connexins and the permeability response to inflammatory stimuli were studied in cultured human pulmonary endothelial cells. Prolonged treatment with thrombin, lipopolysaccharide, or pathological cyclic stretch increased levels of mRNA and protein for the major connexin, connexin43 (Cx43). Thrombin and lipopolysaccharide both increased intercellular communication assayed by transfer of microinjected Lucifer yellow. Although thrombin decreased transendothelial resistance in these cells, the response was attenuated by pretreatment with the connexin inhibitor carbenoxolone. Additionally, the decreases of transendothelial resistance produced by either thrombin or lipopolysaccharide were attenuated by reducing Cx43 expression by siRNA knockdown. Both carbenoxolone and Cx43 knockdown also abrogated thrombin-induced phosphorylation of myosin light chain. Taken together, these data suggest that increased lung vascular permeability induced by inflammatory conditions may be amplified via increased expression of Cx43 and intercellular communication among pulmonary endothelial cells.

## Introduction

Vascular permeability plays a key role in the pathogenesis of inflammatory diseases such as acute respiratory distress syndrome (ARDS) and sepsis, which together account for 400,000 deaths in the United States per year [1], [2]. Compromised endothelial barrier function and resultant permeability during inflammatory injury causes fluid accumulation in the tissues [3]. Edema in the lung impairs the transfer of oxygen to and removal of carbon dioxide from the blood [4]. Further, increased vascular permeability allows destructive proteases, proinflammatory cytokines, and leukocyte-attracting chemokines into the tissues along with the fluid [5]–[7]. Unfortunately, while edema is a dangerous clinical problem associated with severe mortality and morbidity, no effective mitigating therapy exists [3]. A better understanding of vascular permeability will allow us to treat ARDS-associated edema.

Various cellular components have been identified that are critical for maintaining the integrity of the pulmonary endothelial barrier, including components of the tight junctions, adherens junctions, and the actin-based cytoskeleton [8], [9]. A third class of intercellular junctions (the gap junction) is also present linking endothelial cells, but its importance in the pulmonary vascular barrier has not been extensively studied.

Gap junctions contain intercellular channels that allow the exchange of ions and secondary signaling molecules between adjacent cells; they can also form “hemichannels” that allow passage of these permeants between the cytosol and extracellular space [10]. Connexins are the subunit proteins that form gap junctions. Six connexins oligomerize to form a hemichannel, and two hemichannels dock to form a complete gap junction intercellular channel [10]. Several connexins (including Cx43, Cx40, and Cx37) are expressed in endothelial cells, but their roles in the function of the endothelium are not well-understood.

Emerging evidence does suggest that connexins may regulate indices of inflammatory signaling such as adhesion molecule expression and leukocyte infiltration. For example, in an atherosclerosis model of LDL receptor-deficient mice, Cx43 depletion reduced numbers of atherosclerotic lesions and inflammatory cells [11]. In another atherosclerotic model, apolipoprotein E-deficient mice, Cx37 knockout increases atherosclerotic lesions by enhancing recruitment of monocytes and macrophages [12]. Similarly, endothelial deletion of a third connexin, Cx40, increased VCAM-1 expression with concomitant increases in monocyte adhesion [13].

These data suggest that connexins may regulate inflammation, though it has not been studied whether they may do so in the lung, where it may mediate the pathogenicity of ARDS.

Given the close connection between inflammatory signaling and permeability, we hypothesized that connexins may modulate vascular permeability in response to barrier disruptive and pro-inflammatory stimuli. To test this hypothesis, we investigated the modulation of Cx43 expression by inflammatory stimuli in pulmonary endothelial cells. We examined three different kinds of stimuli: (1) thrombin, a barrier-disruptive protease that is activated in ARDS [14], [15]; (2) lipopolysaccharide (LPS), an inflammatory mediator of sepsis, and (3) cyclic stretch. We also measured the permeability response to inflammatory stimuli following pharmacological inhibition of connexin function or specific knockdown of connexin expression. Overall, our data suggest that connexin expression is upregulated by pro-inflammatory or barrier-disruptive agonists, and this may amplify permeability induced by inflammatory conditions in the vascular endothelium.

## Materials and Methods

### Cell culture and reagents

Human pulmonary artery endothelial cells (HPAECs), human pulmonary microvascular endothelial cells (PMVECs), and cell culture medium were obtained from Lonza Inc. (Allendale, NJ, USA), and cells were used at passages 5–8. Di-Phospho-MLC antibodies were obtained from Cell Signaling (Beverly, MA, USA). Beta-actin and Connexin 43 antibodies were obtained from Sigma (St Louis, MO, USA). Unless specified, biochemical reagents were obtained from Sigma.

### Measurement of transendothelial electrical resistance

Cellular barrier properties were analyzed by measurements of transendothelial electrical resistance (TER) across confluent HPAEC monolayers using an electrical cell-substrate impedance sensing system (Applied Biophysics, Troy, NY, USA) as previously described [16], [17].

### Immunofluorescence

Endothelial monolayers were subjected to immunofluorescence staining as described previously [18], [19]. Briefly, HPAECs were grown to confluence on glass cover slips, subjected to appropriate treatment, fixed in 3.7% formaldehyde (10 min., room temp), permeabilized using 0.25% Triton X-100 in PBS (30 min., room temp.), blocked in 3% BSA in TBS-T (30 min., room temp.), and probed with appropriate primary and secondary antibodies, and finally mounted on slides. Slides were analyzed using a Nikon video imaging system (Nikon Instech Co., Tokyo, Japan). Images were processed with Image J (National Institutes of Health, Bethesda, MD, USA) and Adobe Photoshop 7.0 (Adobe Systems, San Jose, CA, USA) software as described [18], [19].

### Immunoblotting

After stimulation, cells were lysed, and protein extracts were separated by SDS-PAGE, transferred to PVDF membrane, and probed with specific antibodies as previously described [20], [21]. Band intensities were quantified by scanning membrane on a Biorad PharosFX Plus Molecular Imager (Bio-Rad; Hercules, CA) and determining the densitometry using Quantity One software (Bio-Rad; Hercules, CA). Background densitometry was subtracted from densitometry of bands of interest before further analysis of data.

### Real-time RT-PCR

After stimulation, cells were collected using Trizol from Ambion (Carlsbad, CA). cDNA was synthesized using Superscript III Cells Direct cDNA Synthesis System from Invitrogen (Carlsbad, CA). Real-time RT-PCR was performed using LightCycler 480 SYBR green PCR master mix kit according to manufacturer’s specifications (Roche; Indianapolis, IN). The amplification conditions were: pre-incubation: 95°C 5 min.; amplification: 45 cycles of the following: 95°C 10 s, 62°C 10 s, 97°C 10 s; melting curve: 95°C 5 s, 65°C 1 min. 97°C (+0.11°C/s); cooling: 40°C 10 s. We used the following primers: Cx43 forward 5′-CGCCTATGTCTCCTCCTGGGTA-3′; Cx43 reverse 5′-TCTGCTTGAAGGTCGCTGGTC–3′; GAPDH forward 5′-ATGGCAAATTCCATGGCACCG-3′; GAPDH reverse 5′- TCGCTCCTGGAAGATGGTGAT-3′).

### Dye transfer assay

After cells were grown to confluence on glass coverslips, gap junction function was measured by micro-injecting single cells with Lucifer yellow (LY) dye and counting the spread of dye to other cells in a cell monolayer. Using a picospritzer (model PLI-188), single cells were microinjected using a micropipette containing 6% LY dye in 150 mM LiCl. Five min. after withdrawing micropipette from cell, the cells were photographed and the cells with fluorescent dye were counted as previously described [22].

### Cyclic Stretch

Cells were grown to confluence on Bioflex plates with elastic wells. Using a FX-4000T Flexcell Tension Plus system (Flexcell International, Hillsborough, NC) equipped with 25 mm BioFlex Loading station, cells were exposed to pathological (18% linear elongation) or physiological (5% linear elongation) cyclic stretch as previously described [23]. Control BioFlex plates with static EC culture were placed in the same cell culture incubator and processed similarly to cyclic stretch-preconditioned cells. At the end of experiment, cell lysates were collected for Western blot analysis or qRT-PCR.

### Small interfering RNA transfection

To reduce the content of endogenous connexin 43 (Cx43), cells were treated with gene-specific small interfering (si) RNA duplexes. Predesigned standard purity siRNA sets (*Homo sapiens*) were purchased (Santa Cruz Biotechnology; Dallas, TX), and transfection of ECs with siRNA was performed as previously described [20],[24]. After 48 hr of transfection, cells were used for experiments or harvested for Western blot verification of specific protein depletion. Nonspecific, nontargeting siRNA (Dharmacon, Lafayette, CO) was used as a control treatment.

### Statistical Analysis

Data are presented as the mean±SE of at least three independent experiments. Stimulated samples were compared to controls by two-tailed, unpaired t-tests. p<0.05 was considered statistically significant.

## Results

### Inflammatory mediators increase Cx43 expression and function

The role of connexins in the maintenance of the endothelial barrier was studied in Human Pulmonary Artery Endothelial Cells (HPAECs). Consistent with previous observations [35], we found that these cells produced high levels of Cx43 and little or no detectable Cx37 and Cx40 (data not shown). We investigated Cx43 expression and function in response to chemical and mechanical ARDS-associated barrier disruptive interventions.

**Figure 1:**
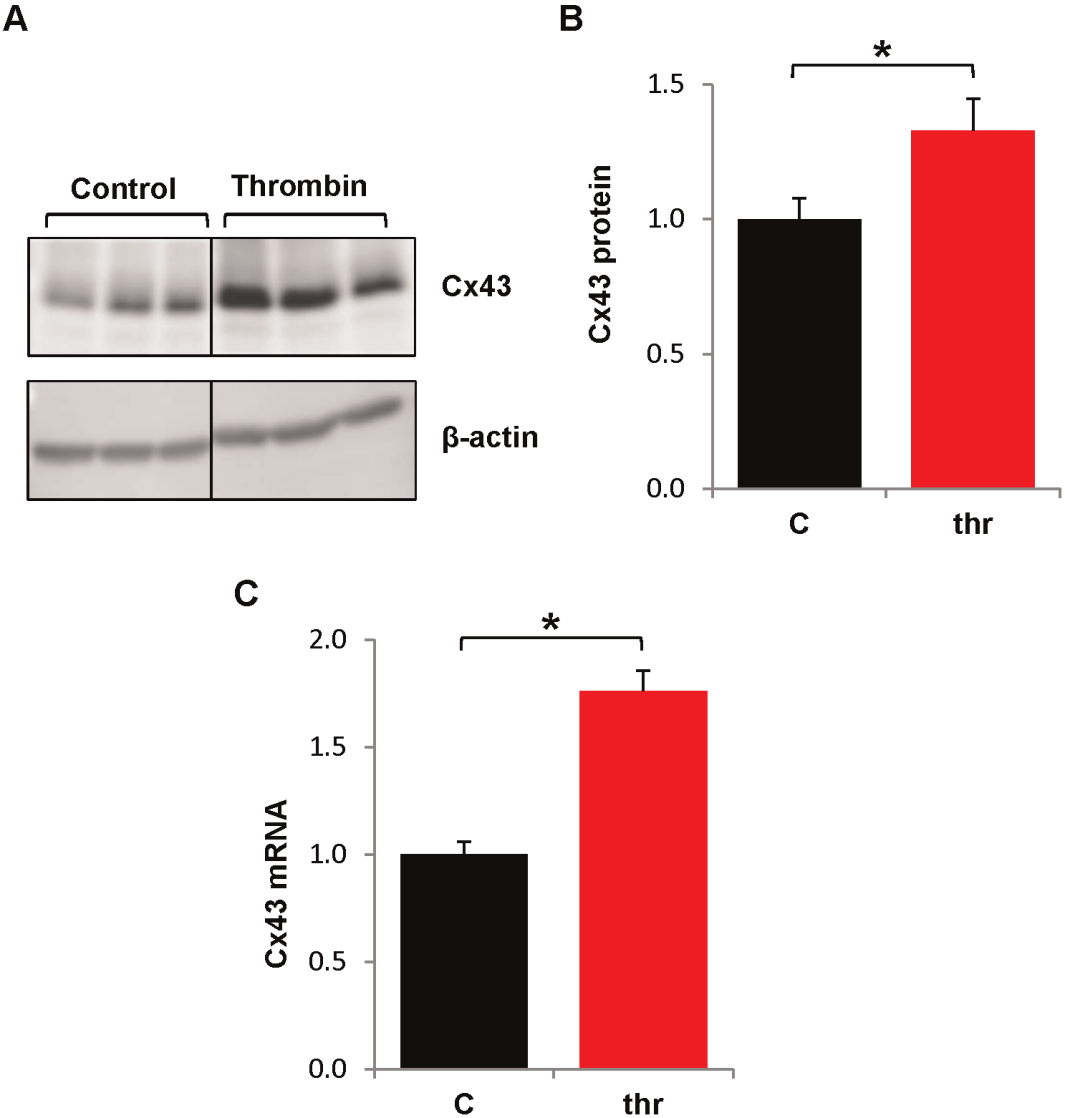
Chronic thrombin treatment increases Cx43 protein expression. HPAECs were left untreated or treated hourly with thrombin (0.5 U/mL) over 6 hr. and Cx43 protein and mRNA were measured. A.) Representative immunoblot shows Cx43 protein levels in thrombin (thr) or control (C) HPAECs. Panel images for control and thrombin bands were recombined from images generated from a single membrane of a Western blot. B.) Graph shows densitometric analysis of Cx43 protein expression. Cx43 protein levels were normalized to β-actin levels and divided by the average control value. *, p<0.05, n = 9. C.) Cx43 mRNA levels were measured by real-time RT-PCR and normalized to GAPDH mRNA and divided by the average control value. *p<0.05, n = 14.

First, we investigated whether Cx43 expression was affected by thrombin. Because thrombin is short-acting (permeability effects begin to abate within 15 min.) and protein synthesis takes hours, thrombin treatments were given repeatedly over 6 hr to mimic the repeated influx of thrombin during chronic inflammation. Cx43 was detected by immunoblotting in control or thrombin-treated HPAECs (**Fig. 1A, B**) Chronic thrombin increased Cx43 protein levels by more than 30% (**Fig. 1B**). When Cx43 mRNA levels from similarly treated cultures were assessed by real-time RT-PCR, we found that chronic thrombin increased Cx43 mRNA ∼75% (**Fig. 1C**).

**Figure 2:**
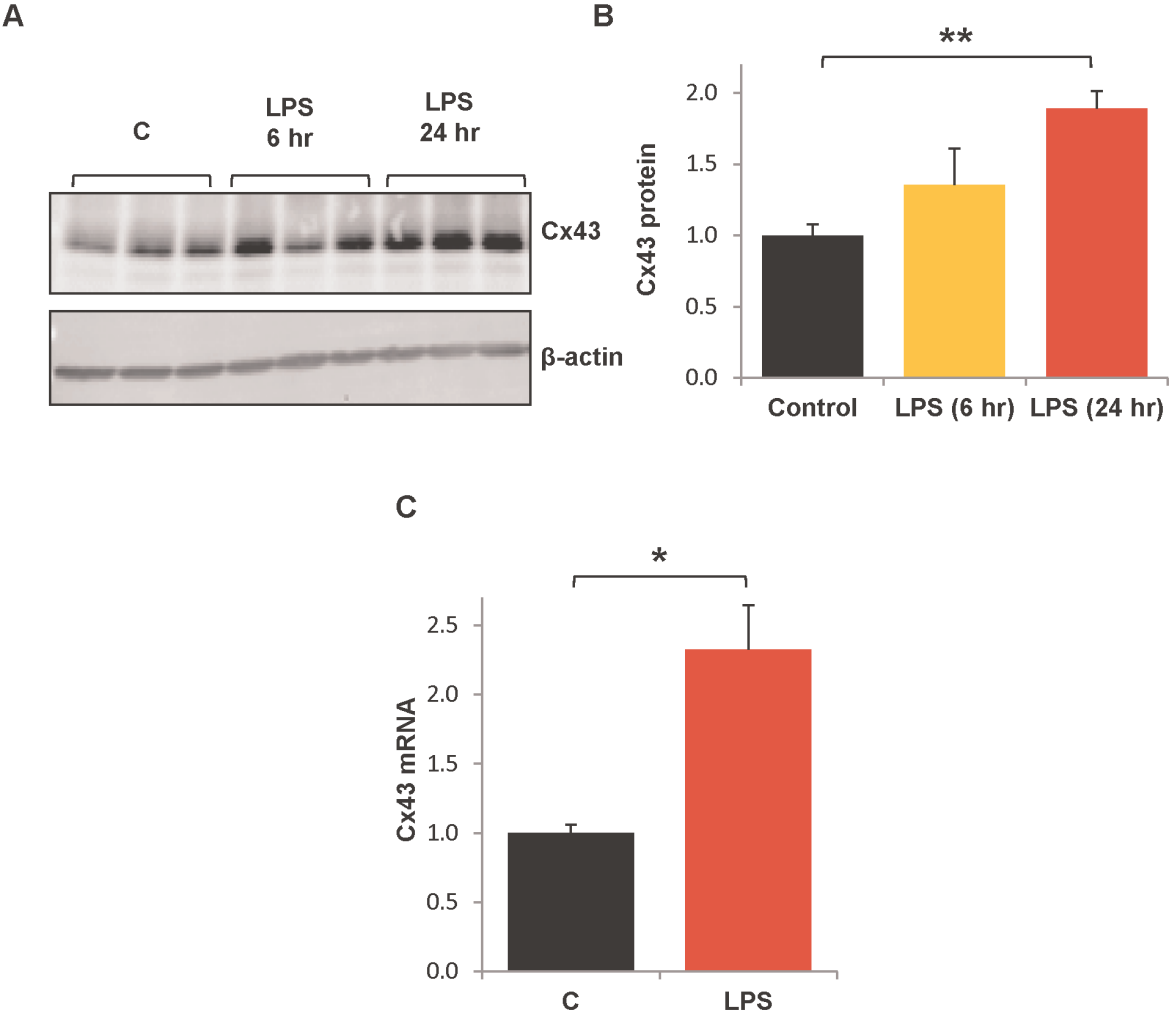
LPS increases Cx43 expression. HPAECs were treated with LPS (200 ng/mL) for 6 hr or 24 hr and collected for Western Blot or qRT-PCR analyses. A.) A representative Western blot is shown. B.) Cx43 protein expression was normalized to β-actin and expressed relative to control. **, p<0.001. n = 6 for control, n = 3 for LPS (6 hr), n = 7 for LPS (24 hr). C.) Real-time RT-PCR analysis was performed on cells treated with LPS (24 hr) or control cultures. Cx43 mRNA levels were normalized to GAPDH mRNA. *, p<0.05, n = 14. C = control.

We also investigated whether Cx43 expression was affected by LPS. HPAECs were treated with LPS for 6 or 24 hr and then collected for Western blot analysis. Although levels appeared to be modestly increased at 6 hr (**Fig. 2**), they did not differ significantly from controls (**Fig. 2B**). After 24 hr of treatment, Cx43 protein levels were significantly increased to about twice baseline levels (**Fig. 2A, B**). Further, Cx43 mRNA expression was more than doubled after 24 hr LPS exposure (**Fig. 2C**).

**Figure 3:**
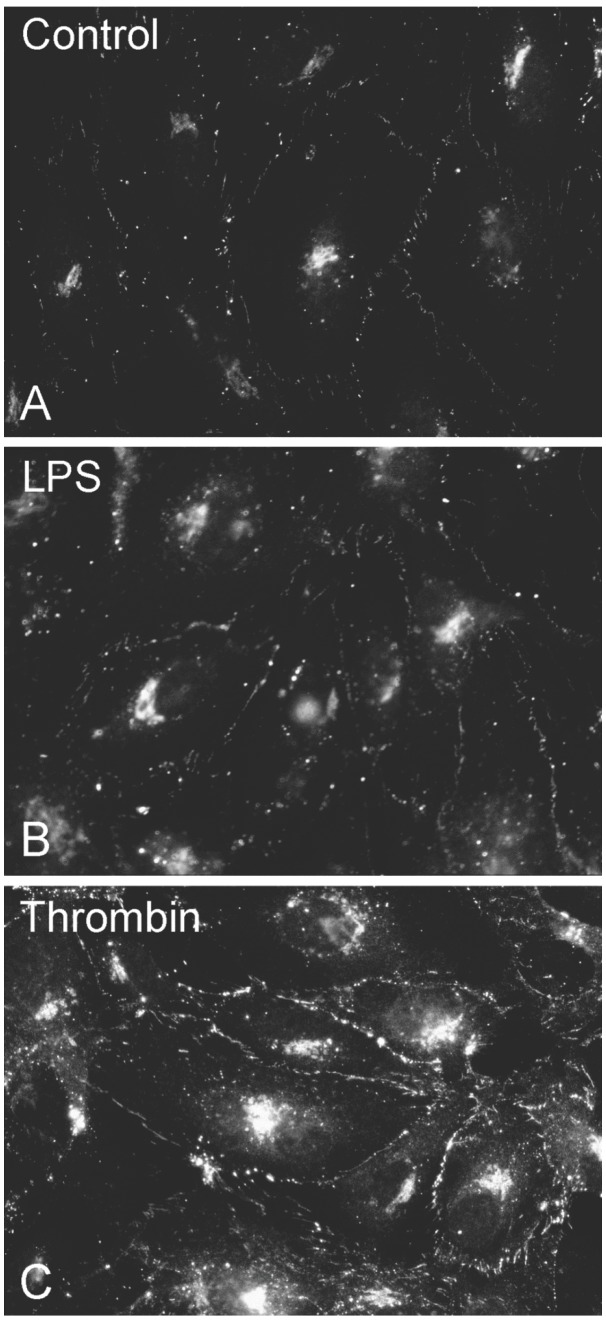
Cx43 distribution between appositional membranes and cytoplasm is unaffected by thrombin or LPS treatment. HPAECs were grown to confluence on glass cover slips and left untreated (A, Control) or treated with LPS (B, 200 ng/mL for 24 hr) or chronic thrombin (C, 0.25 U/mL hourly for 6 hrs). Then, cells were subjected to indirect immunofluorescent staining using a Cx43 antibody.

We also examined the cellular localization of Cx43 in control HPAECs or cell cultures treated with chronic thrombin or LPS by indirect immunofluorescence microscopy (**Fig. 3**). In both control and treated cells, immunoreactive Cx43 showed similar distributions, within the cytoplasm (likely within the biosynthetic/secretory pathway) and at appositional membranes (in locations consistent with gap junction plaques).

**Figure 4:**
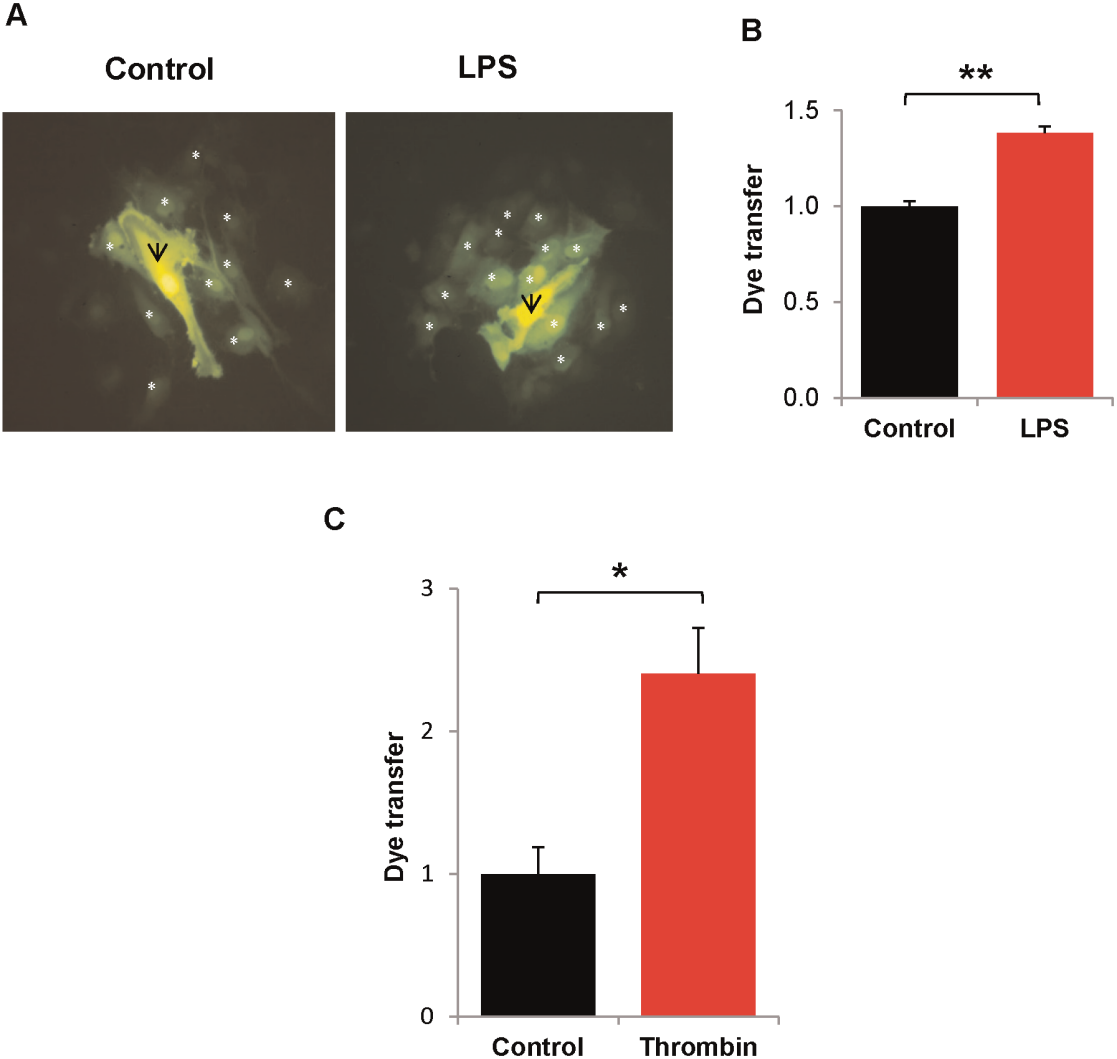
LPS and chronic thrombin treatments increase intercellular communication. HPAECs were grown to confluence on glass cover slips and treated with LPS (200 ng/mL for 24 hr) or chronic thrombin (0.25 U/mL; once every hr for 6 hr.) Single cells were injected with Lucifer yellow for 1 min., and cells were photographed after an additional 5 min. A.) Photomicrographs show representative fluorescence images of an injected cell and its neighbors after injection of Lucifer yellow in a control culture or a culture treated with LPS. The injected cell is marked with a black arrow; neighboring cells which show dye transfer are marked with a white asterisk. B.) Graph shows the composite data for Lucifer yellow dye transfer in control or LPS-treated HPAECs. Values shown are normalized by dividing the number of dye-containing neighbors by the average value obtained by injections in control cultures. **, p<0.001. n = 4. C.) Graph shows the composite normalized data for Lucifer yellow dye transfer in control or thrombin-treated HPAECs. *, p<0.05, n = 3.

The impact of the inflammatory mediators on connexin function was also examined. Cells were similarly treated with LPS or chronic thrombin, and then gap junction-mediated intercellular communication was studied by micro-injecting Lucifer yellow and determining the number of dye-containing neighbors (**Fig. 4**). Both treatments led to significant increases in the extent of dye coupling. We also examined the potential effects of thrombin and LPS on connexin hemichannel function by assaying the uptake of extracellular Lucifer yellow; we found little uptake in control cultures (incubated in the presence or absence of Ca^2+^) or in cultures treated with the inflammatory mediators (data not shown).

### Pathological Cyclic stretch increases Cx43 expression

**Figure 5:**
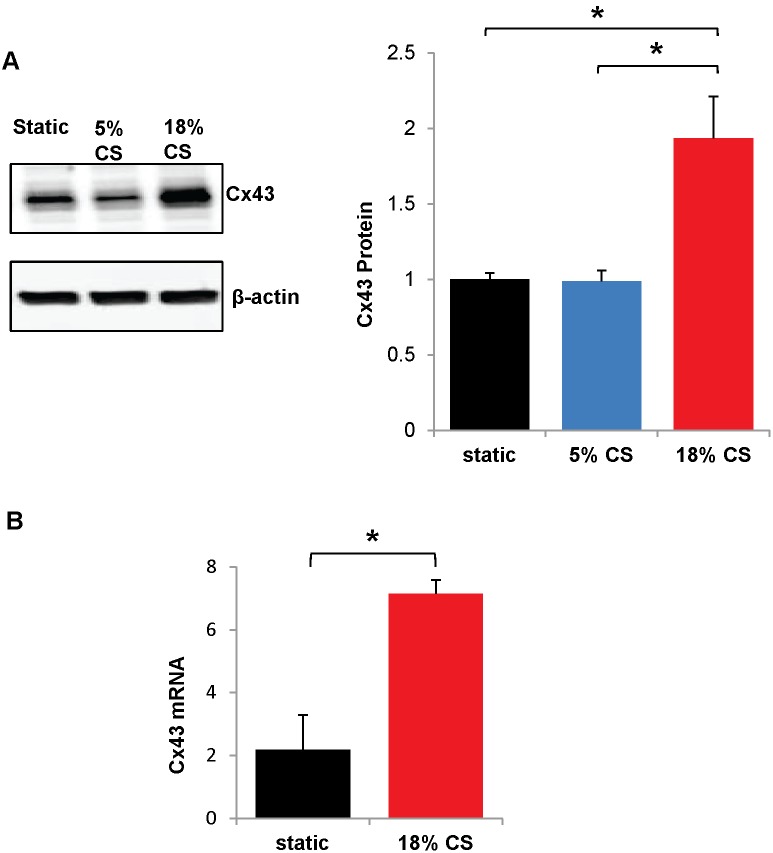
Cyclic stretch increases Cx43 expression. HPAECs were grown to confluence on flexible Bioflex plates; subjected to pathological cyclic stretch (18% elongation), physiological cyclic stretch (5% elongation), or static stretch (0% elongation) for 24 hr.; and Cx43 protein or mRNA was measured. A.) 18% cyclic stretch (18% C.S.) is compared to physiological (5%) stretch control (5% C.S.) and static stretch control. A representative Western blot is shown. Cx43 is normalized to β-actin housekeeping protein levels and expressed relative to static stretch. *, p<0.05. n = 7 for 18% stretch and static stretch; n = 5 for 5% stretch. B.) To measure Cx43 mRNA, cells were subjected to 18% cyclic stretch or static stretch control as before and samples were analyzed by qRT-PCR. Cx43 mRNA was normalized to GAPDH housekeeping mRNA. n = 6. *p<0.05.

Pathological cyclic stretch is a mechanical stimulus which mimics ventilator induced lung injury and causes endothelial barrier disruption. Since the chemical stimuli, LPS and thrombin, increased Cx43 expression, we tested whether cyclic stretch also affected Cx43. HPAECs were grown to confluence on 6-well plates with flexible bottoms (Bioflex plates), and subjected to cyclic stretch for 24 hr. Both a physiological (5% cyclic stretch) and a static (no stretch) control were used [25], [26]. Cx43 protein was doubled by 18% cyclic stretch as compared to static stretch or 5% cyclic stretch controls (**Fig. 5A**). To evaluate whether cyclic stretch regulates Cx43 transcription, mRNA levels of Cx43 were measured by real-time RT-PCR. Cx43 mRNA was more than tripled by 18% cyclic stretch with respect to static control (**Fig. 5B**).

### The gap junction inhibitor, carbenoxolone, attenuates the permeability response to thrombin and inhibits myosin light chain (MLC) phosphorylation

**Figure 6:**
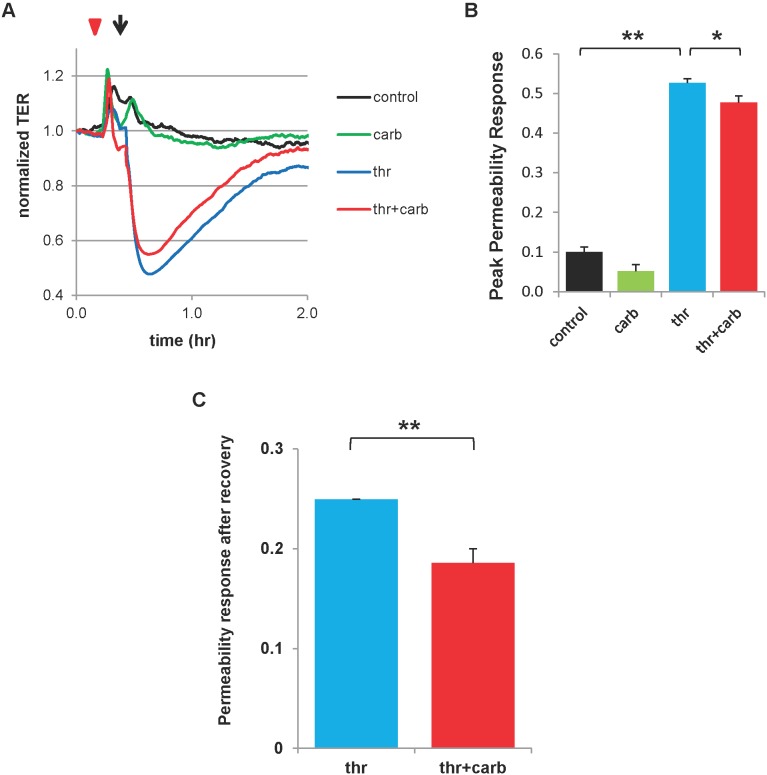
Gap junction inhibitor carbenoxolone attenuates permeability response to thrombin. Confluent HPAECs were treated with carbenoxolone (carb) for >5 min (red triangle; 100 µM) followed by treatment with thrombin (arrow; 0.5 U/mL) and transendothelial resistance (TER) was measured. TER was recorded prior to any treatments and continued an additional 1.5 hr after treatments. A.) Representative TER traces are shown. B.) Peak permeability response is given as [1–(minimum normalized TER)], where normalization is to TER at the time point just prior to the first treatment. **, p<0.001; *, p<0.05; n = 13 for control, n = 11 for carb alone, and n = 15 for thr alone and thr+carb. C.) Permeability response after recovery is given as (1–normalized TER), where TER is taken at the time point in recovery where the thrombin alone group recovers to reach a permeability of <0.25; this is roughly 1 hr after thrombin treatment. As before, TER is normalized to the time point just prior to the first treatment. *p<0.001, n = 13 for each group. Carb = carbenoxolone, Thr = thrombin.

To screen for a contribution of connexins to agonist-induced endothelial permeability changes, we pre-treated human pulmonary artery endothelial cells (HPAECs) with the non-selective gap junction inhibitor, carbenoxolone, prior to application of permeability-inducing thrombin. Paracellular permeability was measured by monitoring transendothelial resistance (TER), which is inversely proportional to the permeability of the monolayer. Treatment with carbenoxolone alone had no significant effect on TER as compared to control (untreated) monolayers (**Fig. 6A**). As expected, thrombin induced a dramatic fall in TER that gradually returned to near baseline levels over ∼1.2 hr. Pretreatment with carbenoxolone modestly attenuated the peak reduction in TER induced by thrombin (**Fig. 6A, B**) while enhancing the recovery to baseline more than 25% (**Fig. 6A, C**).

**Figure 7:**
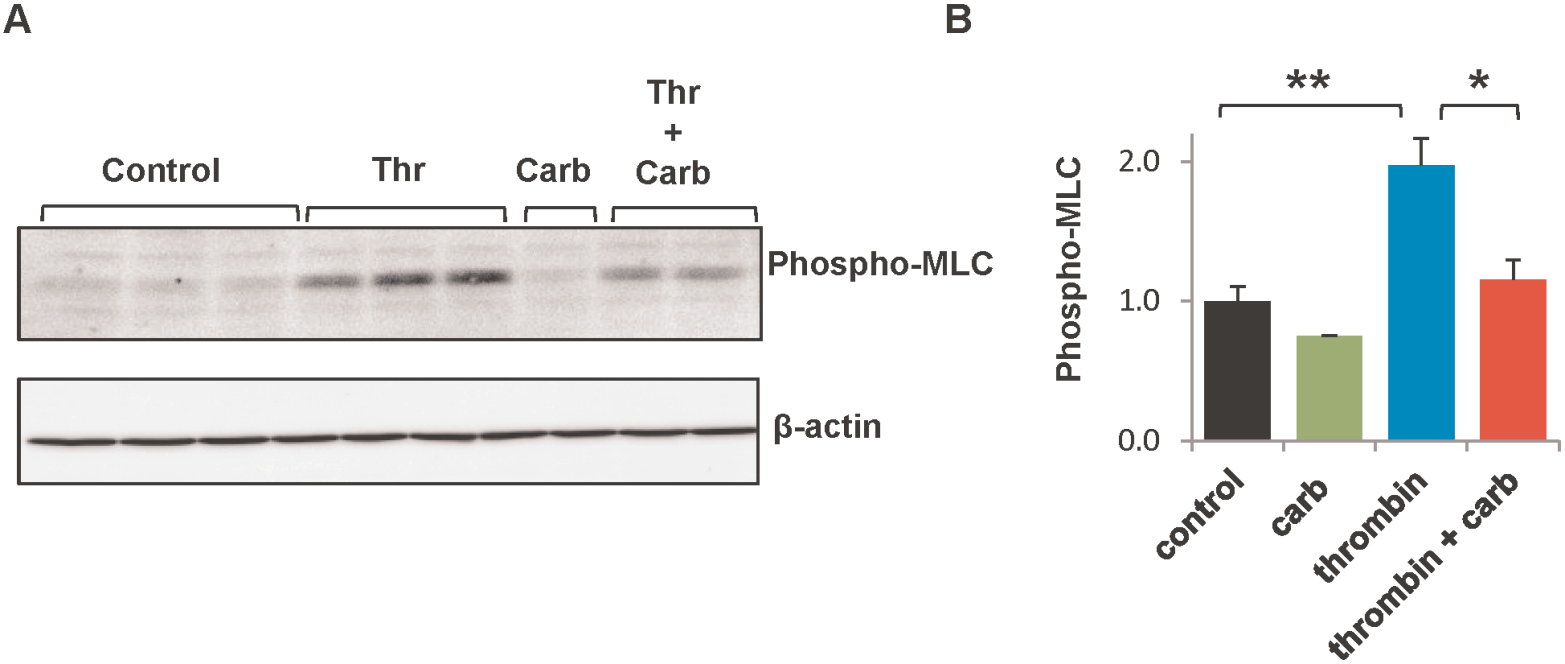
Gap junction inhibitor carbenoxolone attenuates thrombin-induced MLC phosphorylation. HPAECs were treated with carbenoxolone for >5 min followed by thrombin treatment (0.5 U/mL; 30 min) and collected for Western Blot. A.) Representative Western Blot is shown. B.) Phospho-MLC protein was normalized to β-actin relative to control. **, p<0.001. *, p<0.05. n = 5 for control (C), thrombin alone (thr), and thrombin+carbenoxolone (thr+carb) groups. n = 1 for carbenoxolone (carb).

MLC phosphorylation regulates actin-mediated cell contraction, and thus is a major mechanism underlying endothelial permeability [8]. Therefore, we also examined the effects of carbenoxolone on thrombin-induced MLC phosphorylation in HPAECs. Control HPAEC cultures and cultures treated with carbenoxolone alone exhibited a modest amount of MLC phosphorylation (**Fig. 7A**). As anticipated, thrombin induced a major increase in the abundance of phospho-MLC (**Fig. 7A**). This increase was essentially completely abolished by pre-treatment with carbenoxolone. Densitometric analysis of multiple similar experiments showed that thrombin increased levels of phospho-MLC by approximately 2-fold, and that carbenoxolone pre-treatment dramatically reduced phospho-MLC to levels ∼15% greater than controls (**Fig. 7B**).

### SiRNA-Cx43 knockdown attenuated thrombin- and LPS-induced permeability

**Figure 8:**
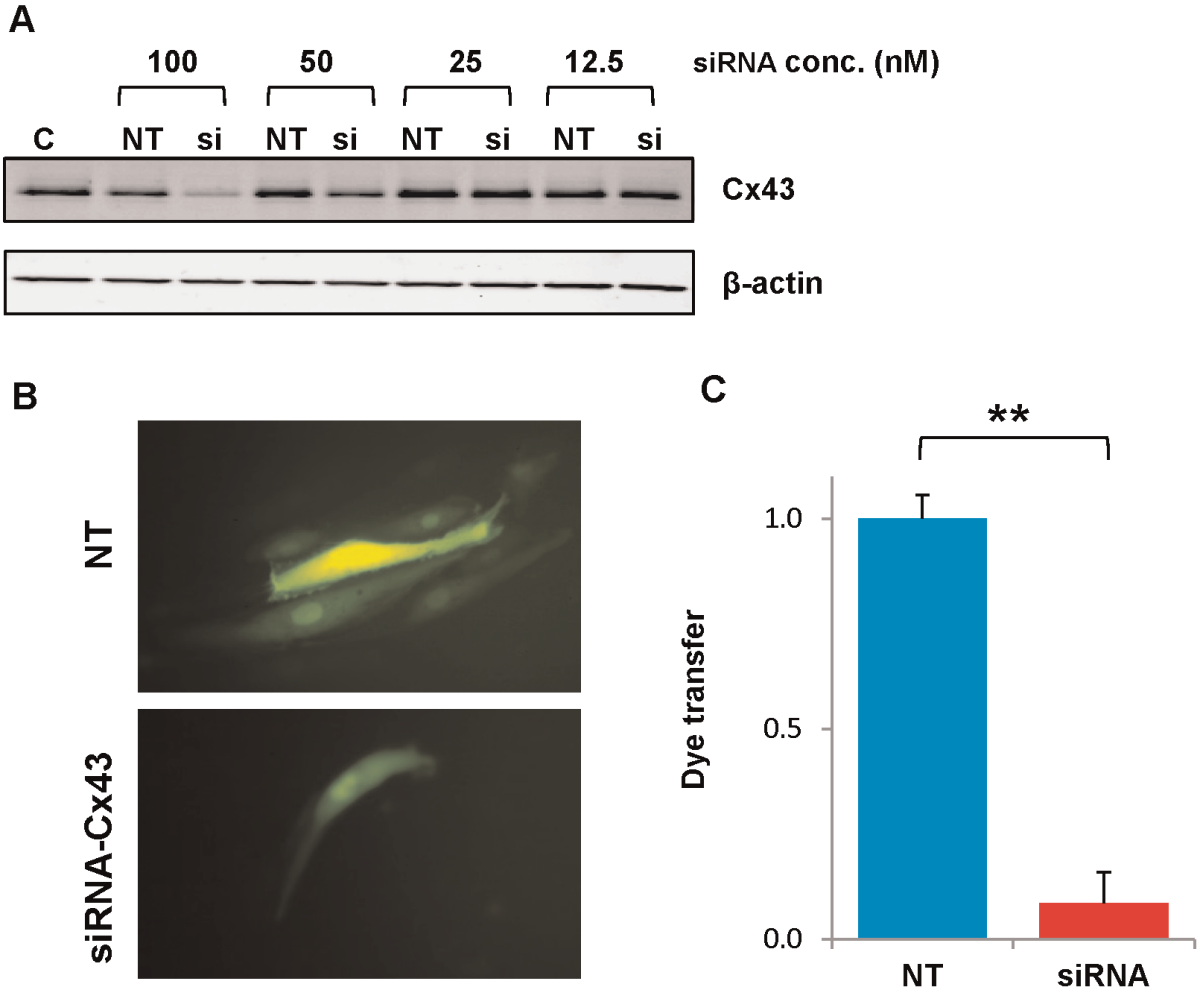
Transfection with siRNA effectively reduces expression and function of Cx43 in HPAECs. Cultures of HPAECs were transfected with Cx43 or control (non-targeting) siRNA 48 hrs prior to various analyses. A.) Western Blots show levels of Cx43 in untreated HPAECs (C) or in cells transfected with different concentrations of non-targeting (NT) or Cx43 (si) siRNA. B.) Fluorescence microscope images show HPAECs micro-injected with Lucifer yellow after transfection with non-targeting (NT) or Cx43 (siRNA-Cx43) siRNA. C.) Graph shows the degree of dye transfer in cultures of HPAECs microinjected with Lucifer yellow after transfection with non-targeting (NT) or Cx43 (siRNA-Cx43) siRNA. Dye transfer was normalized by dividing the number of dye containing neighbors by the average value obtained in NT transfected controls. **, p<0.001, n = 6 for each group.

Although carbenoxolone inhibits gap junction channels, this drug also has many other effects [27]. Therefore, we sought to test a more specific inhibitor. Because Cx43 is the major gap junction protein expressed by HPAECs and many other kinds of cultured endothelial cells [28], we used transfections with siRNAs targeting Cx43 to reduce connexin levels and function in our cells. Immunoblots showed that the greatest reductions of Cx43 levels were achieved using a 100 nM concentration of siRNA (**Fig. 8A**); therefore, this concentration was used for all subsequent experiments. We also tested the effects of siRNA on gap junction function. HPAECs were transfected with siRNA-Cx43 or a control (non-targeting) siRNA, and intercellular communication was tested 48 hr. later by micro-injection of a gap junction permeant tracer, Lucifer yellow (**Fig. 8B**). Control cultures (transfected with non-targeting siRNA) showed extensive dye transfer of Lucifer yellow (on average to 7 neighboring cells) (**Fig. 8B, C**). Dye transfer was essentially abolished (reduced by more than 90% from control levels) in HPAECs transfected with siRNA-Cx43 (**Fig. 8B, C**).

**Figure 9:**
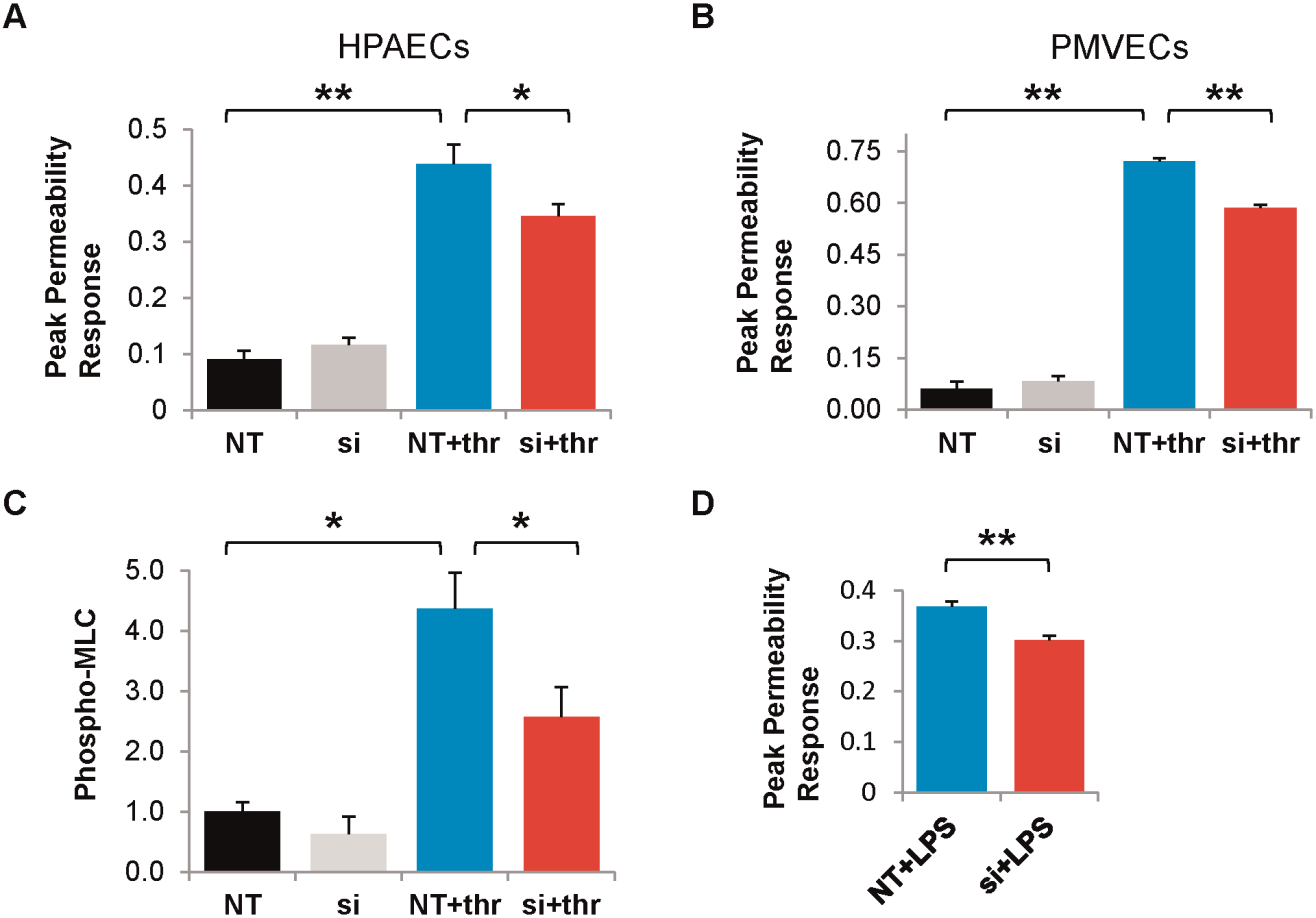
Cx43 knockdown attenuates permeability induced by thrombin or LPS. (**A.**) Graphs show the normalized permeability responses in HPAECs that were transfected with non-targeting (NT) or Cx43 (si) siRNAs for 48 hr and then left untreated or treated with 0.5 U/mL thrombin for 3 hr. (NT+thr and si+thr). Permeability response is given as (1– minimum normalized TER), where normalization is to TER at time point just prior to the first treatment. **, p<0.001. *, p<0.05. n = 8 for NT or siRNA alone; n = 11 for NT+thr group; n = 13 for si+thr group. (**B.**) Graphs show the normalized permeability responses in Pulmonary Microvascular Endothelial Cells (PMVECs) that underwent identical siRNA transfection and thrombin treatment. **, p<0.001. n = 3 for NT+thr and si+thr groups; n = 4 for NT group; n = 3 for si groups. (**C.**) HPAECs were transfected with Cx43 or control siRNA for 48 hr, treated with thrombin (0.5 U/mL; 30 min) and collected for Western Blot. Phospho-MLC protein was normalized to β-actin relative to control. *, p<0.05. n = 6 for each condition. (**D.**) Graphs show the normalized permeability responses in siRNA-transfected HPAECs that had been treated with 200 ng/mL lipopolysaccharide (LPS) for 24 hr. **, p<0.001. n = 7 for each group. NT = non-targeting siRNA, si = siRNA-Cx43. Thr = thrombin.

We tested the impact of siRNA-mediated knockdown of Cx43 on the permeability response induced by thrombin. Transfection of HPAECs with siRNA-Cx43 significantly reduced the peak permeability response (by>25%) as compared to cells transfected with non-targeting siRNA (**Fig. 9A**). To determine the generality of this observation in different lung endothelial tissue types, we performed a similar experiment using pulmonary microvascular endothelial cells (PMVECs) (**Fig. 9B**). Thrombin also induced a major fall in transendothelial resistance (increase in permeability) in the PMVECs; siRNA-Cx43 knockdown reduced this effect by ∼20% (**Fig. 9B**).

The role of Cx43 in our thrombin model of permeability was further elucidated by investigating the effects of Cx43 knockdown on thrombin-induced MLC phosphorylation, as MLC phosphorylation is known to drive the primary mechanism underpinning thrombin-induced permeability [8]. HPAECs were transfected with siRNA-Cx43 or non-targeting (NT) siRNA, treated with thrombin, and then collected for Western blot. As expected, thrombin induces a four-fold increase in phospho-MLC. Cx43 depletion reduced this phospho-MLC response by more than 40%, demonstrating that Cx43 plays an integral role in MLC phosphorylation (**Fig. 9C**). While there appeared to be a slight attenuation in MLC phosphorylation in cells treated with siRNA-Cx43 alone compared to NT alone, this trend was not statistically significant (**Fig. 9C**).

To examine the generality of our permeability findings in different models of permeability, we examined the effects of LPS, which also produced a strong permeability response (**Fig. 9D**). This response was significantly reduced by transfection with siRNA-Cx43 as compared to non-targeting siRNA (**Fig. 9D**). Taken together, our data suggest that Cx43 may globally influence the permeability response in lung endothelium.

## Discussion

The main findings of this study are that the expression of the gap junction protein, Cx43, is increased by a diverse set of ARDS-related inflammatory stimuli in pulmonary endothelial cells and that Cx43 influences the magnitude of the barrier disruption that these stimuli cause.

We found that two inflammatory mediators, thrombin and LPS, increased Cx43 protein and mRNA levels as well as the extent of intercellular communication. The effects of LPS on connexin levels and gap junction-mediated coupling have been studied in several different systems and appear to have different consequences depending on cell type or connexin expressed. Our findings are consistent with some of the studies of endothelial cells and other cells expressing Cx43. Injection of LPS into rats [29] or intra-tracheal administration in mice [30] cause increases in Cx43 expression in the lungs and kidneys, but its expression is down-regulated in other tissues like the heart [31]. LPS has also been found to induce Cx43 expression in leukocytes [32]. In some endothelial cells, LPS reduces intercellular coupling, but this effect is likely due to their predominant expression of Cx40 (which is affected differently and may be decreased by inflammation) [33]–[35]. The effects of thrombin on endothelial connexins have not been extensively investigated. Thrombin can induce the internalization of gap junctions [36]. Both thrombin and LPS can cause the opening of connexin “hemi-channels” [37], [38], but we did not see any significant uptake of dye tracers in either untreated or treated HPAECs.

Cyclic stretch can mimic the mechanical trauma induced by lung ventilation [28], [29]. We found that this stimulus also increased Cx43. Similar increases have previously been observed in other cells types from tendon, bone, and heart (reviewed by [39]).

Our data demonstrate a novel role for gap junctions in the lung endothelium. Inhibition of connexin function via carbenoxolone attenuated thrombin-induced permeability. Cx43 knockdown attenuated both thrombin- and LPS-induced permeability. Connexin inhibition or knockdown markedly attenuated thrombin-induced MLC phosphorylation, suggesting connexins may regulate actin-mediated cell contraction, a major pathway underlying permeability.

From our observations, we have assembled a model illustrating that inflammatory stimuli induce expression of Cx43, elevating the abundance of gap junctions and the extent of intercellular communication, and thereby exacerbating the increases in endothelial permeability. Recent reports showing attenuation of acid- and thrombin-induced permeability in the lung microvasculature by Cx43-blocking peptides support this model [40], [41]. However, in those whole animal studies, other cell types (e.g., pulmonary epithelial cells) likely also contribute to permeability. Another report found increased trans-endothelial permeability of rat lung endothelial cells treated with gap junction inhibitors alone [42]. Their data contrast with our study, which shows no permeability effect of gap junction inhibition in the absence of an inflammatory mediator. The differences in results may derive from use of a different model species or use of different gap junction inhibitors (which may have nonspecific effects). Moreover, they studied gap junction inhibition in the absence of a stimulus which tests the role of *basal* intercellular signaling, while we focused on gap junctional communication *in response to stimulus*. It is entirely possible that the type of signaling (and the signaling molecules passing through the gap junction channels) may be different in quiescent and inflammatory conditions.

The role of gap junctions in endothelial paracellular permeability is not well-understood. Rather, adhesion complexes such as the tight junctions and adherens junctions are established as the regulators of permeability at the membrane, restricting paracellular flux by sealing the intercellular gaps through adhesion of apposing junctional proteins [9]. It is unlikely that the gap junctions contribute a significant increment in adhesive function (beyond the cadherin-based adherens junctions). Our studies also exclude a significant role of connexins as opening hemichannels. Therefore, the connexins likely participate in the permeability response to inflammatory stimuli through their function in intercellular communication, by facilitating the intercellular exchange of signals that coordinate or enhance the response. However, the specific signaling pathway(s) and gap-junction permeant signaling molecules involved are unknown. Gap junction channels allow the intercellular passage of many small signaling molecules, including calcium and other ions, IP3, ATP, and cyclic nucleotides [43]. Calcium ions may be one of the best candidates, since Parthasarathi *et al.* showed that Cx43 facilitates the spread of calcium signaling in lung microvasculature [40]. Recent evidence shows that microRNA may also pass through gap junctions, suggesting it as an additional candidate [44].

Our data imply that inflammatory stimuli increase the number of gap junction channels based on our observed increase in the abundance of Cx43-containing gap junctions and intercellular dye transfer. This contrasts with the fact that LPS induces the formation of intercellular gaps (which reflect increased paracellular permeability). It is possible that the connexins may be redistributed away from these intercellular gaps in order to maintain the function of gap junctions. Alternatively, it may be that the large upregulation in connexin expression (doubling for LPS, thrombin, and pathological cyclic stretch) increases the surface connexin density to such a degree that overall gap junction communication is enhanced despite the loss of contact between cells at areas where intercellular gaps have formed.

While our study demonstrates the role of Cx43 in regulation of pulmonary endothelial permeability, the role of other connexins found in different endothelial cells (such as Cx40 and Cx37) in permeability regulation is not known. In atherosclerotic models, Cx40 and Cx37 are anti-inflammatory [12], [13] while Cx43 is pro-inflammatory [11]. As paracellular permeability is linked to inflammation, this suggests that Cx40, Cx37, and Cx43 may play contrasting roles in its regulation. These connexins form channels that differ in physiological properties (such as permeant size and charge selectivity) and regulation which may contribute to contrasting roles in the regulation of inflammation and permeability [43], [45]. Analysis of Cx40 and Cx37 functions in different vascular endothelial beds deserves further investigation, although poor expression in tissue culture for these isotypes remains a technical challenge.

In conclusion, our results show that Cx43 exacerbates the permeability response of lung endothelium to a diverse set of ARDS-related inflammatory stimuli. These data suggest that gap junctions may play a key role in the development of edema in the lung. Therefore, modulation of connexin expression and function in lung vascular endothelium may represent a novel potential therapeutic approach for reducing the severity of pulmonary edema and ARDS.
